# Retraining walking adaptability following incomplete spinal cord injury

**DOI:** 10.1038/s41394-017-0003-1

**Published:** 2017-12-14

**Authors:** Emily J. Fox, Nicole J. Tester, Katie A. Butera, Dena R. Howland, Martina R. Spiess, Paula L. Castro-Chapman, Andrea L. Behrman

**Affiliations:** 1 Department of Physical Therapy, University of Florida, Gainesville, FL USA; 2 Brooks Rehabilitation Clinical Research Center, Jacksonville, FL USA; 3 Brain Rehabilitation Research Center, Malcom Randall VAMC, Gainesville, FL USA; 4 Department of Occupational Therapy, University of Florida, Gainesville, FL USA; 5 Department of Neurological Surgery, Kentucky Spinal Cord Injury Research Center, University of Louisville, Louisville, KY USA; 6 Robley Rex VA Medical Center, Louisville, KY USA; 7 Hocoma AG, Volketswil, Switzerland; 8 James A. Haley VAMC, Tampa, FL USA; 9 Department of Psychiatry and Behavioral Neuroscience, University of South Florida, Tampa, FL USA

## Abstract

**Introduction:**

Functional walking requires the ability to modify one’s gait pattern to environmental demands and task goals—gait adaptability. Following incomplete spinal cord injury (ISCI), gait rehabilitation such as locomotor training (Basic-LT) emphasizes intense, repetitive stepping practice. Rehabilitation approaches focusing on practice of gait adaptability tasks have not been established for individuals with ISCIs but may promote recovery of higher level walking skills. The primary purpose of this case series was to describe and determine the feasibility of administering a gait adaptability retraining approach—Adapt-LT—by comparing the dose and intensity of Adapt-LT to Basic-LT.

**Case presentation:**

Three individuals with ISCIs (>1 year, AIS C or D) completed three weeks each (15 sessions) of Basic-LT and Adapt-LT. Interventions included practice on a treadmill with body weight support and practice overground (≥30 mins total). Adapt-LT focused on speed changes, obstacle negotiation, and backward walking. Training parameters (step counts, speeds, perceived exertion) were compared and outcomes assessed pre and post interventions. Based on completion of the protocol and similarities in training parameters in the two interventions, it was feasible to administer Adapt-LT with a similar dosage and intensity as Basic-LT. Additionally, the participants demonstrated gains in walking function and balance following each training type.

**Discussion:**

Rehabilitation that includes stepping practice with adaptability tasks is feasible for individuals with ISCIs. Further investigation is needed to determine the efficacy of Adapt-LT.

## Introduction

Recovery of walking function is a primary goal and focus of rehabilitation for individuals with incomplete spinal cord injuries (ISCIs) [1]. To walk safely within the home and community, individuals must generate a basic stepping pattern and also modify one’s gait pattern to changing environmental demands (e.g., obstacles, speed changes) and task goals [2]. Locomotor training (LT) is an established gait rehabilitation approach for improving walking function in individuals with ISCIs [3–5]. However, this approach emphasizes practice of a basic stepping pattern and the majority of studies have focused outcomes on '‘steady state’' walking conditions [6]. Few reports of individuals with ISICs have addressed retraining of gait adaptations necessary for walking in varied environments—walking adaptability.

In our search of the literature, only two reports have focused on strategies for retraining gait adaptability skills following ISCI [7, 8]. In a case series of four adults with ISCIs, Musselman et al. (2009) compared overground skill training that included walking on different surfaces, varying environments, and walking with a secondary task such as carrying an object, to training that focused on '‘steady state’' treadmill walking with body weight support. Participants improved following both forms of training and the skill training was reported to be as effective as the treadmill-based intervention [7]. Subsequently, Yang and colleagues [8] conducted a clinical trial to compare outcomes following overground training with visually-guided tasks such as stepping over obstacles and on targets ('‘precision’' training) to treadmill-based '‘endurance’' training. Although individuals demonstrated improvements following both types of training, the greatest gains in walking speed and endurance were achieved following rehabilitation involving repetitive stepping practice on a treadmill. Interestingly, the authors reported that repetitive treadmill stepping involved speeds and step counts three-times faster and greater during each session than during overground training [8]. These differences in speeds and amount of practice (i.e., dosage) were likely important factors in the study outcomes since it is well-established that amount of practice is critical for inducing motor re-learning and plasticity [9, 10]. Additionally, stepping speed is an important training parameter since faster speeds induce greater and more reciprocal lower extremity muscle activation during treadmill walking in adults with SCI [11] and training at higher speeds is associated with improvements in treadmill walking speed [12]. Thus, it may be a detrimental trade-off if dosage and intensity are compromised when adaptability tasks are incorporated into gait rehabilitation.

Spinal cord injury (SCI) gait rehabilitation principles that emphasize repetitive stepping practice and speed [13] are based on animal studies in which the spinal neural networks involved in basic stepping patterns contribute to recovery and have been shown to respond to training [14, 15]. In contrast, walking adaptations such as visually-guided limb movements (e.g., stepping over obstacles) and stepping backward (e.g., to back up to a chair) involve greater cortical activation [16, 17]. Overall, since functional walking requires activation of *both* spinal *and* cortical neural networks [18], it may be that gait rehabilitation should incorporate *all* of these features—repetitive stepping practice, increased speeds, as well as practice of adaptability tasks.

Although SCI gait rehabilitation that incorporates all of these features has not been established, different types of adaptability training have been successfully applied to adults post-stroke, and in other populations [19–22]. For instance, overground adaptability training that included an obstacle course to simulate daily walking and walking exercises such as speed changes was shown to reduce falls and improve obstacle avoidance skills in older adults [19, 20]. Recent studies in adults post-stroke have demonstrated that gait adaptability training on a treadmill with augmented virtual targets and obstacles (C-Mill) improves walking speed, balance, and increases performance of adaptability tasks, which is associated with reduced attentional demands [21, 22].

Overall, these prior studies suggest that gait rehabilitation with adaptability tasks may be beneficial for individuals with ISCIs, but there may be challenges to achieving a sufficiently high dosage (number of steps) and intensity during adaptability training. Furthermore, adaptability training could be particularly difficult to implement for individuals with ISCIs due to common impairments such as severe bilateral weakness and spasticity. To address these challenges, a gait rehabilitation approach, referred to as Adapt-LT was developed. Adapt-LT includes basic stepping practice (Basic-LT), but also implements repetitive practice of gait adaptability tasks—obstacle negotiation, backward walking, and speed changes. Therefore, the goals of this case series were to describe the Adapt-LT approach and determine the feasibility of delivering Adapt-LT at a dosage and intensity similar to training with Basic-LT. We specifically focused on whether a similar number of steps, stepping speeds, and self-reported exertion levels could be achieved for both Adapt-LT and Basic-LT. Walking function and balance also were assessed at the end of each intervention.

## Case Presentation

### Participants

This case series was conducted at the Malcom Randall Veterans Affairs Medical Center Brain Rehabilitation Research Center in Gainesville, Florida. Institutional and federal regulations concerning ethical use of human volunteers were followed; all protocols were approved by the University of Florida and Veterans Affairs Medical Center (Gainesville, FL). Participants provided informed consent prior to enrollment. Eligibility criteria included ≥ 18 years old with a singular, motor ISCI (≥6 months post-injury), medically stable, discharged from physical therapy, and able to ambulate at speeds of ≥0.3 m/s at the time of enrollment.

Three adult males (26–77 years) with ISCIs (durations > 18 months) were enrolled. Descriptive information was obtained through review of medical records, participant self-report, and assessment at time of enrollment. A licensed physical therapist completed all clinical assessments. The International Standards for Neurological Classification of Spinal Cord Injury (ISNCSCI) American Spinal Injury Association Impairment Scale (AIS) was used to classify each participant’s neurologic level of injury (Table 1) [23]. Strength in five key muscle groups was assessed using the AIS guidelines for Lower Extremity Motor Scores (Table 2) [24].

**Table 1:** Participant characteristics

Participants	SCI01	SCI02	SCI03
Age (gender)	66 years old (male)	67 years old (male)	26 years old (male)
Mechanism of injury	Non-traumatic	Non-traumatic; surgery	Traumatic; car accident
Type of injury lesion	UMN	UMN	UMN and LMN
Neurologic injury level	T4	C6	L2
AIS classification	C	D	C
Time post-injury	108 months	25 months	18 months
Rehabilitation history	Home health PT	Inpatient rehab PT and OT	Inpatient rehab PT and OT
	Inpatient rehab PT and OT	Outpatient PT and OT	Outpatient PT and OT
Gait status	Limited household	Community	Limited household
Assistive devices	RW	None	RW and bilateral AFOs
Primary mode of mobility	Wheelchair	Ambulation	Wheelchair
Home environment and level of independence	Lives alone in house; independent	Lives with spouse in house; independent	Lives with girlfriend in apartment; independent
Employment	Retired	Retired	Unemployed since car accident
Other activities and participation	Swimming regular exercise	Travels frequently	Regular walking practice and exercise

*UMN* upper motor neuron lesion, *LMN* lower motor neuron lesion, *C* cervical, *T* thoracic, *L* lumbar (number that follows refers to vertebral level), *PT* physical therapy, *OT* occupational therapy, *RW* Rolling walker, *AFO* Ankle-foot orthosis, *AIS* American Spinal Injury Association Impairment Scale

AIS level and classification (level = neurologic level of injury; classifications: C = Incomplete; motor function is preserved below the neurological level and more than half of key muscles below the neurological level have a muscle grade less than 3; D = Incomplete; motor function is preserved below the neurological level and at least half of key muscles below the neurological level have muscle grade greater than or equal to 3) [23]

All case information reflects status at time of enrollment

**Table 2:** Lower extremity motor scores at time of enrollment

Participants	SCI01		SCI02		SCI03	
Total lower extremity motor score	23/50		46/50		22/50	
	R	L	R	L	R	L
Hip flexors	2	4	4	3	5	5
Knee extensors	3	2	5	5	5	5
Ankle dorsiflexors	1	2	5	4	0	0
Long toe extensors	4	3	5	5	0	0
Ankle plantar flexors	1	1	5	5	1	1

Lower extremity motor scores were assessed according to the International Standards for Neurological Classification of Spinal Cord Injury [23]

### Procedures

**Fig. 1:**
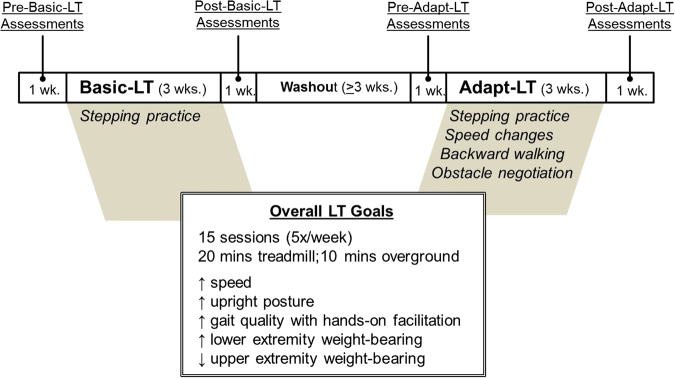
Case series design and training parameters. *LT* locomotor training, *Basic-LT* basic locomotor training, *Adapt-LT* Adapt locomotor training

The participants completed two interventions—15 sessions of Basic-LT, followed by 15 sessions of Adapt-LT. Each intervention consisted of five sessions per week for 3 weeks, with a minimum 3-week wash-out period between interventions (Fig. 1). Basic-LT is an established intervention with which we have experience [3, 25–27]. For that reason and to ensure the safety of each participant, Basic-LT was administered first which allowed baseline training responses to be established. Feasibility outcomes reflecting intervention dose and intensity included step count, participant’s perceived exertion, and training speed. Clinical outcomes to characterize walking function and balance were assessed one week prior to and within one week after the completion of each intervention.

#### Interventions overview

Figure 1 details the intervention timeline and Adapt-LT features. Intervention sessions were 30 min (minimum) in duration and included ~20 min of training on a treadmill, followed by ~10 min of overground practice. Standing breaks were provided as needed and did not count toward the training duration. Practice on the treadmill (Biodex Medical, Shirley, NY) included use of a harness (Robertson Mountaineering, Henderson, NV) and partial body weight support (Robomedica, Culver City, CA). An initial body weight support of <40% was targeted. Training on the treadmill was progressed by increasing speed and lowering body weight support. Manual assistance was provided during training on the treadmill and overground to promote appropriate kinematics. Arm swing was encouraged and treadmill rails were not used. Training overground was completed on a level surface with assistive devices/braces, if needed to assure safe participation. Verbal cues and encouragement, as well as performance and results feedback were provided during training sessions.

#### Basic locomotor training

Basic-LT involved repetitive stepping practice with the goals of increasing speed, promoting gait quality, and enhancing lower extremity weight bearing [3, 13]. Specifically, each participant trained at the fastest speed they could safely achieve and sustain with adequate loading on the lower limbs and use of body weight support ≤40%. Appropriate gait kinematics were promoted during treadmill and overground training by providing verbal cues and manual assistance as needed, at the lower limbs and trunk. During overground training participants were encouraged to walk at their maximal speed.

#### Adapt locomotor training

The goal in developing Adapt-LT was to apply principles of LT such as repetitive stepping, increased training speeds and intensity, and to incorporate practice of adaptability tasks. Adaptability tasks were selected based on several factors: (a) common tasks representing different adaptability domains; [2, 28] (b) tasks that could be practiced both on the treadmill and overground and; (c) tasks known to emphasize different aspects of neuromuscular control such as increased cortical engagement and visuomotor coordination (e.g., obstacle negotiation) or different motor strategies (e.g., backward walking) [16–18].

**Fig. 2:**
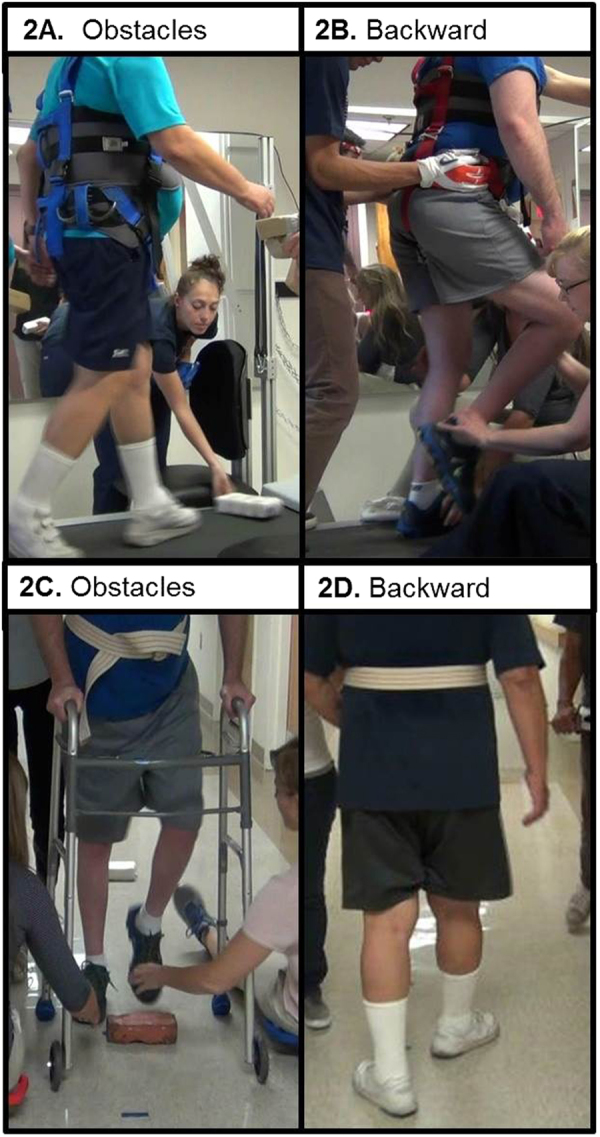
Treadmill and Overground Adapt-locomotor training. **a** Participant SCI01 performing obstacle negotiation on treadmill. **b** Participant SCI02 performing backward walking on treadmill. **c** Participant SCI03 performing obstacle negotiation overground. **d** Participant SCI02 performing backward walking overground. *TM* treadmill, *OG* overground

Thus, based on these factors, Adapt-LT emphasized the same general principles as Basic-LT and also included practice of adaptability tasks (obstacle negotiation, backward walking, and speed changes) (Figs. 1b and 2a–d). During training on the treadmill the goal was to spend a minimum of 5 min on each adaptability task (Fig. 2): 1. *Obstacle negotiation* -Obstacles were delivered bilaterally and included foam blocks and boxes of variable sizes (height range: 5–14 cm; width range: 20–39 cm; depth range: 5–24 cm). Progression of the task included increasing obstacle frequency, varying rates of obstacle delivery, and increasing obstacle size. 2. *Speed changes*-Speed changes consisted of abruptly and unexpectedly changing from faster speeds to and from slower speeds. 3. *Backward walking -*For progression, backward walking speed was increased and body weight support was decreased. During overground training, participants performed the same tasks and the goal was to spend a similar amount of time on each task.

### Training parameters and intervention feasibility

Feasibility was determined based on the successful completion of the Adapt-LT protocol and focused on comparisons of dosage (amount of steps) and intensity (speeds, perceived exertion) parameters across Basic-LT and Adapt-LT. Additionally, these parameters were assessed because of their importance in motor relearning (i.e., rehabilitation) and association with improved outcomes in studies of walking function after SCI [9–11].

#### Amount of stepping practice

During training on the treadmill, steps were counted for 15–30 s at each speed and the total number of steps was estimated for each session. During training overground, the total number of steps was counted by an assistant.

#### Maximal treadmill training speeds

The average maximal treadmill training speed was determined for sessions 11–15 based on the highest speed sustained for at least 30 s or, as in the case of speed changes during Adapt-LT, a speed achieved at least twice during the session.

#### Training intensity

For each training bout, participants were asked to report their exertion level using the 20-point Borg Rating of Perceived Exertion Scale [29]. Reported Rating Scale scores were averaged within and across the 15 sessions for Basic and Adapt-LT.

### Clinical walking function and balance outcomes

Clinical assessments of walking function and balance were administered before and after 15 sessions of Basic-LT and 15 sessions of Adapt-LT (Fig. 1). The 10 meter walk test (10 MWT), [30, 31] Timed Up and Go (TUG) [32] and Spinal Cord Injury Functional Ambulation Profile (SCI-FAP [33] were used to characterize walking function; and the Mini Balance Evaluations Systems Test or MiniBESTest (MBT [34] and Activities-Specific Balance Confidence Scale (ABC) [35, 36] were administered to assess balance. The 10 MWT was used to measure fastest comfortable gait speed. [30, 31] The TUG assesses the capacity to perform transitional movements such as rising from a chair and turning around [32]. The SCI-FAP assesses walking function during seven walking tasks (e.g., walking around obstacles, stepping over obstacles) [33]. The MBT assesses dynamic balance during sitting, standing, and stepping tasks [34] while the ABC was used to assess self-reported balance confidence during a variety of gait activities [35, 36].

### Outcomes

#### Overview

All three participants completed the training protocols and exceeded the goal of training for 30 min per session; the average total training time was 40 min for Basic-LT and 41 min for Adapt-LT. SCI01 did not complete the Basic-LT post-assessment due to illness and holiday travels; therefore, outcome scores for the Adapt-LT pre-assessment were used for the Basic-LT post-assessment values.

**Fig. 3:**
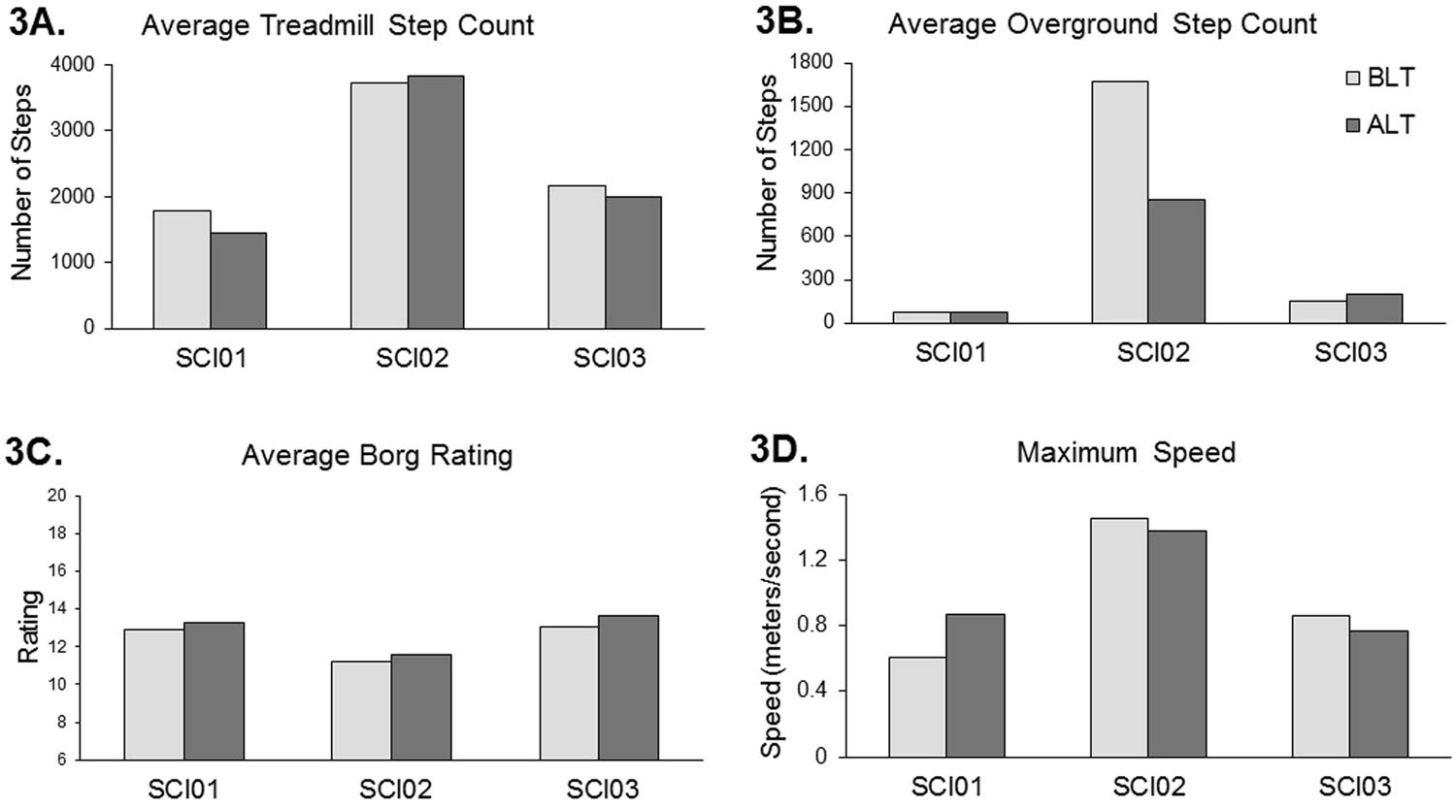
Training parameters for the three participants (SCI01-SCI03) during Basic- and Adapt-locomotor training. **a** Average number of steps during training on the treadmill. **b** Average number of steps during training overground. **c** Average Borg Ratings of Perceived Exertion during training. **d** Maximum training speeds. *BLT* Basic locomotor training, *ALT* Adapt locomotor training

The overall outcomes indicate that the Adapt-LT was feasible and was administered at a similar dosage and intensity as Basic-LT (Fig. 3). The participants, all of whom had chronic injuries (>18 months duration) and two individuals required the use of a wheelchair for mobility, demonstrated improvements in walking function and balance. Individual outcomes are described below and are summarized in Fig. 3 and Table 3a, b. Results that exceed the established Minimal Clinically Important Difference (MCID) for individuals with SCIs also are reported.

**Table 3:** Summary of clinical outcomes

(a) Walking function												
	TUG (s)				SCI-FAP (score/2100)				Gait Speed (m/s)			
	Pre-BLT	Post-BLT	Pre-ALT	Post-ALT	Pre-BLT	Post-BLT	Pre-ALT	Post-ALT	Pre-BLT	Post-BLT	Pre-ALT	Post-ALT
SCI01	35.97	43.75	43.75	33.53	156.34	181.92	181.92	166.84	0.35	0.34	0.34	0.36
SCI02	13.16	14	11.21	10.2	15.89	11.57	9.2	13.17	1.18	1.56	1.29	1.48
SCI03	59.44	59.4	48.46	45.19	967.9	706.7	433.41	425.23	0.28	0.31	0.33	0.44

(b) Balance								
	MBT (score/28)				ABC (%)			
	Pre-BLT	Post-BLT	Pre-ALT	Post-ALT	Pre-BLT	Post-BLT	Pre-ALT	Post-ALT
SCI01	7	8	8	10	50.94	41.25	41.25	54.06
SCI02	21	26	24	24	Not tested	57.5	66.88	67.5
SCI03	6	7	7	9	33.75	34.38	40.63	45

Walking Function: *TUG* Timed Up and Go, *SCI-FAP* Spinal Cord Injury Functional Ambulation Profile, Gait Speed obtained from 10 Meter Walk Test

Balance: *MBT* Mini Balance Evaluation Systems Test (MiniBESTest), *ABC* Activities-Specific Balance Confidence Scale

*Pre-BLT* pre Basic-LT, *Post-BLT* post Basic-LT, *Pre-ALT* pre Adapt-LT, *Post-ALT* post Adapt-LT

SCI01 did not complete the post Basic-LT assessment due to illness and holiday travels; therefore, outcome scores for the Pre Adapt-LT assessment were used as the Post Basic-LT values. SCI02 did not complete the ABC prior to Basic-LT; therefore, the overall change was obtained from comparing post Basic-LT scores to post Adapt-LT scores

#### Participant outcomes

##### SCI01

*Dosage and Training Intensity*. SCI01 achieved an average of 1790 steps during Basic-LT on the treadmill and 1453 steps during Adapt-LT. During each session of overground training an average of 72 steps were practiced for both types of training. Average maximal treadmill speeds were higher for Adapt-LT (Basic-LT = 0.61 m/s, Adapt-LT = 0.87 m/s) and Borg ratings of exertion were similar for both training types (Basic-LT = 12.92, Adapt-LT = 13.31). SCI01’s walking pattern was characterized as stiff and he had particular difficulty in flexing his lower extremity joints. Therefore, the treadmill environment and hands-on assistance may have been helpful in achieving sufficient stepping practice. Additionally, the adaptability tasks may have encouraged less stiff movements (e.g., lower extremity flexion to step over obstacles or use of a different movement pattern to step backward) and enabled training at higher speeds.

*Basic and Adapt-LT Outcomes*. Following 3 weeks of Basic-LT, no gains in walking function or balance were seen for SCI01. In contrast, after Adapt-LT, SCI01 showed a reduced TUG time (∆ = ↓10.22 s), increased MBT score (∆↑ = 2), and increased ABC score (∆ = ↑12.81%).

*Overall Outcomes*. At the conclusion of the study, SCI01 demonstrated increased walking function (∆TUG = ↓2.44 s) and increased balance (∆MBT = ↑3; ∆ABC = ↑3.12%) compared to baseline. The changes, however, did not exceed MCID values for individuals with SCIs. Prior to his enrollment, SCI01 could not stand without support for >5 s. It is, therefore, note-worthly that following the completion of both interventions he stood without support for >1 min. SCI01 reported that he perceived improvement in his walking function and general mobility. He stated that he now believed getting better was possible, whereas he did not believe this was possible before training.

##### SCI02

*Dosage and Training Intensity*. SCI02 achieved similar average treadmill step counts during both types of training (Basic-LT = 3718 steps, Adapt-LT = 3822 steps), but performed nearly double the number of steps overground during Basic-LT (Basic-LT = 1675 steps, Adapt-LT = 865 steps). For this individual who walked at a faster speed, the adaptability tasks likely took a relatively greater time to complete. His average maximal training speeds and Borg ratings were similar for both training types (Basic-LT = 1.45 m/s, Adapt-LT = 1.38 m/s; Borg ratings: Basic-LT = 11.20, Adapt-LT = 11.59).

*Basic and Adapt-LT Outcomes*. SCI02 demonstrated greater improvements following Basic-LT. His gains in gait speed (∆ = ↑0.38 m/s) exceeded the MCID value for individuals with SCIs [37]. He also achieved an increased MBT score (∆ = ↑5) as well as improved his SCI-FAP score (∆ = ↓4.32).

*Overall Outcomes*. At the conclusion of the study, SCI02 demonstrated an increase in walking function (∆gait speed = ↑0.30 m/s; ∆TUG = ↓3.16 s) and increased balance (∆MBT = ↑3; ∆ABC = ↑10%) compared to baseline. SCI02 did not complete the ABC prior to Basic-LT; therefore, this overall change was obtained from comparing post Basic-LT scores to post Adapt-LT scores. The increase in gait speed exceeded the MCID value (0.13 m/s) for individuals with SCIs [38]. SCI02 anecdotally reported more confidence in community walking; he stated this high confidence contributed to more participation in walking, longer walking distances and durations.

##### SCI03

*Dosage and Training Intensity*. SCI03 performed an average of 2161 steps during each session of Basic-LT on the treadmill and 2006 steps during Adapt-LT. During each session of overground training an average of 147 steps were practiced during Basic-LT and 203 steps for Adapt-LT. Average maximal treadmill speeds were similar (Basic-LT = 0.86 m/s, Adapt-LT = 0.77 m/s) and Borg ratings of exertion also were similar for both training types (Basic-LT = 13.08, Adapt-LT = 13.62).

*Basic and Adapt-LT Outcomes*. SCI03 showed greater positive changes in almost all clinical outcomes following Adapt-LT compared to BLT. He demonstrated increased gait speed (∆ = ↑0.11 m/s), reduced TUG time (∆ = ↓3.27 s), a higher MBT score (∆ = ↑2) and a higher ABC score (∆ = ↑4.38%). In contrast, only the SCI-FAP score (∆ = ↓261.2) showed a greater positive change following Basic-LT. The improvements following Adapt-LT exceeded the MCID value (0.13 m/s) for gait speed in individuals with SCIs [38].

*Overall Outcomes*. At the conclusion of the study, SCI03 demonstrated increased walking function (∆gait speed = ↑0.16 m/s; ∆TUG = ↓14.25 s; ∆SCI-FAP = ↓542.67) and balance (∆MBT = ↑3; ∆ABC = ↑11.25%). The increase in gait speed and decrease in TUG time both exceeded the MCID values for individuals with SCIs (0.13 m/s and 10.8 s, respectively) [38]. Interestingly, SCI03 was previously excluded from enrollment in studies of walking function post SCI due to evidence of peripheral lumbar nerve injury. Due to paralysis of the ankle muscles (Table 2), he completed overground training with ankle-foot orthotics. However, ankle-foot orthotics were not used during training on the treadmill because it was thought that this would alter afferent input associated with training. Thus, training on the treadmill required careful hands-on assistance to assure ankle stability and safety.

## Discussion

The focus of this case series was to describe Adapt-LT and determine the feasibility of administering Adapt-LT at a dosage and intensity similar to Basic-LT. Three adults with chronic ISCIs completed 15-sessions of Adapt-LT and the amount of stepping practice, treadmill speeds, and perceived exertion were similar to Basic-LT. Moreover, outcomes at the conclusion of training indicated that participants improved in walking and balance function, suggesting that ongoing improvements are achievable in individuals with chronic ISCIs. Overall, our goal in developing Adapt-LT was to build off of previous SCI rehabilitation strategies and address the challenges of implementing a gait adaptability intervention with a similar dosage and intensity of Basic-LT [8].

Gait rehabilitation principles applied in the development and administration of Adapt-LT are well-established [3, 13] and focus on training parameters to promote activation of spinal neural networks, induce neural plasticity, and engage supraspinal pathways [16, 39–42]. While activation of these pathways was not assessed, the Adapt-LT emphasized parameters to enhance afferent feedback, repetition, and intensity to promote plasticity [9, 10, 43, 44]. Engagement of supraspinal and visual-motor pathways was emphasized through the demand to negotiate obstacles by adjusting foot trajectory [17]. Further, balance and stepping challenges were incorporated by performance of backward stepping and by inducing speed changes [16].

A recent study of retraining gait adaptability post-ISCI reported potential limitations of their approach were reduced amounts of practice and slower training speeds [8]. To overcome these challenges, we used a treadmill, partial body weight support and hands-on assistance during training. These elements enabled us to safely administer Adapt-LT and provide a similar dosage and training intensity as Basic-LT. Further, use of the treadmill and body weight support reduced fall risk and reduced participants’ fears of falling during practice of challenging adaptability tasks. This was particularly important for SCI01 and SCI03 who had more severe impairments and required a wheelchair for mobility. Training on the treadmill and overground for these two partcipants often utilized three trainers (1 physical therapist and 2 assistants), as well as an assistant to manage the equipment and set-up adaptability tasks (e.g., deliver obstacles on the treadmill). While this amount of assistance and personnel may pose a challenge in clinical settings, new paradigms are emerging for SCI rehabilitation to address issues pertaining to resources and service delivery [45].

Overall, the general training parameters we applied across Basic- and Adapt-LT were consistent with previously-reported studies describing intense SCI gait rehabilitation. Specifically, the duration of training and training speeds used during both interventions were consistent with prior reports [6]. In addition, the amount of steps practiced and training intensities achieved during Adapt-LT were in-line with prior reports of SCI walking adaptability [7, 8]. The participants achieved an average of 1500–4000 steps per session of Adapt-LT, which is consistent with the number of steps practiced during '‘Endurance Training’' (treadmill stepping),’ as reported by Yang et al. (2014). Our participants reported slightly lower levels of perceived exertion (range 11.6–13.6 for Adapt-LT) relative to the ratings reported by Musselman et al. (2009) (range 12.5–17.6 for Skill Training). Additionally, the walking and balance outcomes achieved following each type of training (Basic- and Adapt-LT) and overall were generally consistent with previous research [6, 46]. Comparisons between studies, however, are challenging not only because prior investigations have applied different research designs, but also because details regarding training parameters (e.g., intensity and amount of steps practiced) are rarely reported.

### Limitations

A potential limitation in the design of this case series was the consistent order of the two types of training. Specifically, the Adapt-LT training parameters and outcomes may have been different had Basic-LT not been administered first. However, based on the goals of this case series, Basic-LT was provided prior to Adapt-LT for a variety of reasons. First, since our focus was to establish feasibility of Adapt-LT, we thought it was important to provide each participant with an established intervention to determine their ability to safely participate and identify baseline training responses. The use of only three adaptability tasks—obstacle negotiation, speed changes, and backward walking—also poses another potential limitation. While these tasks are important elements of community mobility, other features such as uneven terrains, doorways, and stairways were not practiced [47]. Finally, although this case series focused on only three individuals, this provided an opportunity to examine individual responses to Adapt-LT in a cohort of participants with heterogeneous injury characteristics and varied walking abilities. In particular, each participant anecdotally reported they felt backward walking practice was particularly challenging and beneficial. Consistent with this, recent reports of backward walking training in individuals with ISCIs suggest this strategy may be useful for promoting recovery of balance and forward walking function. [48, 49]

### Conclusions and future directions

Adapt-LT, a gait rehabilitation intervention focused on repetitive practice of walking and tasks requiring gait adaptability was feasible for the three individuals with chronic ISCIs. In most instances, the training parameters of amount of practice, walking speed, and perceived exertion were consistent across Basic- and Adapt-LT. Although responses to training were varied, gains in walking and balance function were achieved. Overall, the outcomes provide preliminary insight into how individuals with ISCIs may respond to varied forms of training. Future studies are necessary to assess the efficacy of Adapt-LT and to further develop this approach to maximize its potential effectiveness for promoting walking recovery.
